# Supplementary material for: The ubiquitylation of IL-1β limits its cleavage by caspase-1 and targets it for proteasomal degradation
Source: Nat Commun. 2021 May 11;12:2713. doi: 10.1038/s41467-021-22979-3 (PMC8113568; doi:10.1038/s41467-021-22979-3)
Supplement: Supplementary file 3 — Reporting Summary [file 41467_2021_22979_MOESM3_ESM.pdf]

nature research

Corresponding author(s): James Vince and Kate Lawlor

Last updated by author(s): Apr 3, 2021

Reporting Summary

Nature Research wishes to improve the reproducibility of the work that we publish. This form provides structure for consistency and transparency in reporting. For further information on Nature Research policies, see our Editorial Policies and the Editorial Policy Checklist.

Statistics

For all statistical analyses, confirm that the following items are present in the figure legend, table legend, main text, or Methods section.

n/a	Confirmed	
☐	☒	The exact sample size (*n*) for each experimental group/condition, given as a discrete number and unit of measurement
☐	☒	A statement on whether measurements were taken from distinct samples or whether the same sample was measured repeatedly
☐	☒	The statistical test(s) used AND whether they are one- or two-sided<br>*Only common tests should be described solely by name; describe more complex techniques in the Methods section.*
☒	☐	A description of all covariates tested
☒	☐	A description of any assumptions or corrections, such as tests of normality and adjustment for multiple comparisons
☐	☒	A full description of the statistical parameters including central tendency (e.g. means) or other basic estimates (e.g. regression coefficient) AND variation (e.g. standard deviation) or associated estimates of uncertainty (e.g. confidence intervals)
☐	☒	For null hypothesis testing, the test statistic (e.g. *F*, *t*, *r*) with confidence intervals, effect sizes, degrees of freedom and *P* value noted<br>*Give P values as exact values whenever suitable.*
☒	☐	For Bayesian analysis, information on the choice of priors and Markov chain Monte Carlo settings
☒	☐	For hierarchical and complex designs, identification of the appropriate level for tests and full reporting of outcomes
☒	☐	Estimates of effect sizes (e.g. Cohen's *d*, Pearson's *r*), indicating how they were calculated

Our web collection on statistics for biologists contains articles on many of the points above.

Software and code

Policy information about availability of computer code

Data collection	MS/MS spectra were processed with MaxQuant (version 1.5.2.8)<br>Western blot acquisition used Image Lab (Biorad) version 6.1<br>Flow cytometry data was collected using Cell Quest (BD Biosciences) v6.1
Data analysis	Flow cytoemetry data was analyzed by WEASEL version 2.7 (https://frankbattye.com.au/Weasel/index.html) and FlowJo version 10.2<br>Western blot analysis was conducted with Image Lab (Biorad) version 6.1<br>Graphs and error bars were generated and calculated using Prism 7 (version 7.0d) from GraphPad Software

For manuscripts utilizing custom algorithms or software that are central to the research but not yet described in published literature, software must be made available to editors and reviewers. We strongly encourage code deposition in a community repository (e.g. GitHub). See the Nature Research guidelines for submitting code & software for further information.

Data

Policy information about availability of data

All manuscripts must include a data availability statement. This statement should provide the following information, where applicable:

- Accession codes, unique identifiers, or web links for publicly available datasets
- A list of figures that have associated raw data
- A description of any restrictions on data availability

The mass spectrometry proteomics data (Figure 4A and 4B) have been deposited to the ProteomeXchange Consortium via the PRIDE partner repository with the dataset identifier PXD021305 (Project Webpage: http://www.ebi.ac.uk/pride/archive/projects/PXD021305 and FTP Download: ftp://ftp.pride.ebi.ac.uk/pride/data/archive/2021/04/PXD021305). Extracted peak lists were searched against the reviewed Homo sapiens (UniProt, March 2015; https://www.uniprot.org/uniprot/

P10749) database containing murine IL1b sequence. The structure of IL-1beta shown in Figure 4E (PDB: 2MIB) can be found at; https://www.rcsb.org/structure/2mib. Source data are available in the Source Data File associated with this manuscript.

Field-specific reporting

Please select the one below that is the best fit for your research. If you are not sure, read the appropriate sections before making your selection.

☒ Life sciences ☐ Behavioural & social sciences ☐ Ecological, evolutionary & environmental sciences

For a reference copy of the document with all sections, see nature.com/documents/nr-reporting-summary-flat.pdf

Life sciences study design

All studies must disclose on these points even when the disclosure is negative.

Sample size	For in vitro assays no statistical analysis was used to determine sample size, but each experiment was repeated 2-4 times independently to verify the results. The exact number of independent experiments performed for each experiment is indicated in the figures legends. For in vivo experiments power calculations were performed. Sample size was determined to ensure there is 80% power to obtain a statistically significant difference between treatment groups at the 5% significance level (5% = p value 0.05). In vivo experiments were repeated 3 times, generally using 5 mice/per genotype, and the results pooled for presentation.
Data exclusions	Data was excluded where there was technical error (e.g. poor transfection efficiency, poor or unequal protein transfer in immunoblotting, high background levels of cell death in untreated cells).
Replication	All experiments were repeated independently in order to ascertain reproducibility and the number of repeats performed for data presented in each figure panel is clearly stated in the figure legends. In cases where variability was observed more repeats were generated, unless there was obvious technical error.
Randomization	In vitro experimental samples and allocation of mice to treatment groups for in vivo studies were selected at random using numerical animal identifiers. Animal genotype was the only selection criteria employed when allocating experimental groups.
Blinding	In vivo experimental blinding was not necessary as the experiments were coordinated by the researchers but actioned by animal technicians. The animal technicians were not informed of the expected result from any genotype. Technicians monitored all animals and assisted in the harvesting of all samples according to ethical requirements and numerical animal identifiers. The samples were then assayed simultaneously by multiple researchers using only the numerical animal identifiers (i.e. 1 researcher performed ELISAs and 1 performed the immunoblots). For in vitro experiments blinding was not necessary as all samples were analysed simultaneously using identical assay conditions and reagents.

Reporting for specific materials, systems and methods

We require information from authors about some types of materials, experimental systems and methods used in many studies. Here, indicate whether each material, system or method listed is relevant to your study. If you are not sure if a list item applies to your research, read the appropriate section before selecting a response.

Materials & experimental systems

n/a	Involved in the study	
☐	☒	Antibodies
☐	☒	Eukaryotic cell lines
☒	☐	Palaeontology and archaeology
☐	☒	Animals and other organisms
☒	☐	Human research participants
☒	☐	Clinical data
☒	☐	Dual use research of concern

Methods

n/a	Involved in the study	
☒	☐	ChIP-seq
☐	☒	Flow cytometry
☒	☐	MRI-based neuroimaging

Antibodies

Antibodies used	All Western blot antibodies were used at a 1:1000 dilution unless noted otherwise.<br>Western blotting:<br>Mouse β-actin Clone AC-15 (Sigma; A-1978).<br>Pro and mature IL-1β (R&D Systems; AF-401-NA).<br>Pro- and cleaved caspase-1 (Adipogen; AG-20B-0042-C100).<br>Pro-caspase-8 (clone 3B10, in-house).<br>Cleaved caspase-8 Asp387 (Cell Signaling; 9429)<br>NLRP3 (Adipogen; AG-20B-0014-C100).<br>ASC (Santa Cruz Biotechnology; Sc-22514-R).<br>IL-18 (BioVision; 5180R-100), 1:500 dilution used.

	IL-1alpha (Cell Signaling; 9672), 1:500 dilution used.<br>Ubiquitin (Cell Signaling; 3933)<br>Mcl-1 (Cell Signaling; 5453)<br>GSDMD (Abcam; ab209845).<br>K48-linked ubiquitin (Cell Signaling;8081)<br>K63-linked ubiquitin (Cell Signaling; 5621)<br>K11-linked ubiquitin (MABS107-I, clone 2A3/2E6; Merck)<br>Immunopurification (IP):<br>biotin-IL-1beta (Biolegend; 503505), used 4 microgram per IP<br>K48-linked polyubiquitin (05-1307, clone Apu 2; Merck), used 4 microgram per IP<br>K63-linked polyubiquitin (05-1308, clone Apu 3; Merck), used 4 microgram per IP<br>Anti-E25 (IgG isotype control, kindly provided by Genentech), used 4 microgram per IP<br>Cell Sorting:<br>CD11b (BD Biosciences, clone M1/70; 553310), used at 1:400.<br>Ly6G (clone 1A8-Ly6g, Invitrogen; 12-9668-82), used at 1:400.
Validation	Mouse β-actin Clone AC-15 (Sigma; A-1978) validated by the manufacturer (https://www.sigmaaldrich.com/catalog/product/sigma/a1978). Pro and mature IL-1β (R&D Systems; AF-401-NA), validated by the manufacturer (https://www.rndsystems.com/products/mouse-il-1beta-il-1f2-antibody_af-401-na) and by ourselves in this study using relevant gene targeted cells (e.g. see Figure 1C). Pro- and cleaved caspase-1 (Adipogen; AG-20B-0042-C100) validated by the manufacture using gene targeted cells (https://adipogen.com/ag-20b-0042-anti-caspase-1-p20-mouse-mab-casper-1.html/). Pro-caspase-8 (in-house), validated by ourselves using gene targeted cells in PMID:30485804. Cleaved caspase-8 Asp387 (Cell Signaling; 9429) validated by the manufacturer (https://www.cellsignal.com/products/primary-antibodies/cleaved-caspase-8-asp387-antibody-mouse-specific/9429) and ourselves using gene targeted cells in PMID:30485804. NLRP3 (Adipogen; AG-20B-0014-C100) validated by the manufacture using gene targeted cells (https://adipogen.com/ag-20b-0014-anti-nlrp3-nalp3-mab-cryo-2.html).<br>ASC (Santa Cruz Biotechnology; Sc-22514-R) validated by ourselves using gene targeted cells in this study (see Figure 3D). IL-18 (BioVision; 5180R-100) validated in PMID: 24610009. IL-1alpha (Cell Signaling; 9672) validated by the manufacturer (https://www.cellsignal.com/products/primary-antibodies/il-1a-d4f3s-rabbit-mab-mouse-specific/50794?site-search-type=Products&N=4294956287&Ntt=il-1&fromPage=plp). Ubiquitin (Cell Signaling; 3933) validated by the manufacturer (https://www.cellsignal.com/products/primary-antibodies/ubiquitin-antibody/3933).<br>Mcl-1 (Cell Signaling; 5453) validated by the manufacturer (https://www.cellsignal.com/products/primary-antibodies/mcl-1-d35a5-rabbit-mab/5453).<br>GSDMD (Abcam; ab209845) validated by the manufactuer (https://www.abcam.com/gsdmd-antibody-epr19828-ab209845.html).<br>K48-linked ubiquitin (Cell Signaling;8081) validated by the manufacturer (https://www.cellsignal.com/products/primary-antibodies/k48-linkage-specific-polyubiquitin-d9d5-rabbit-mab/8081). K63-linked ubiquitin (Cell Signaling; 5621) validated by the manufacturer (https://www.cellsignal.com/products/primary-antibodies/k63-linkage-specific-polyubiquitin-d7a11-rabbit-mab/5621).<br>K11-linked ubiquitin (MABS107-I, clone 2A3/2E6; Merck) was validated in PMID: 20655260.<br>Biotin-IL-1beta (Biolegend; 503505) validated by ourselves in this study using gene targeted cells (see Figure 7A). K48-linked polyubiquitin (05-1307, clone Apu 2; Merck), K63-linked polyubiquitin (05-1308, clone Apu 3; Merck) and anti-E25 (IgG isotype control, kindly provided by Genentech) validated in PMID: 18724939. CD11b (BD Biosciences, clone M1/70; 553310) validated by the manufacturer (https://www.bdbiosciences.com/us/applications/research/stem-cell-research/mesenchymal-stem-cell-markers-bone-marrow/mouse/negative-markers/fitc-rat-anti-cd11b-m170/p/553310). Ly6G (clone 1A8-Ly6g, Invitrogen; 12-9668-82) validated by the manufacturer (https://www.thermofisher.com/antibody/product/Ly-6G-Antibody-clone-1A8-Ly6g-Monoclonal/12-9668-82).

Eukaryotic cell lines

Policy information about cell lines

Cell line source(s)	Cells were derived by the authors from mice generated by the Walter and Eliza Hall Institute of Medical Research. 293T cells were purchased from ATCC (https://www.atcc.org/Products/Cells_and_Microorganisms/Cell_Lines.aspx)
Authentication	Genotype and species were authenticated in house using PCR based methods and/or next-generation sequencing.
Mycoplasma contamination	Passaged cell lines used in this study (293Ts) were tested for mycoplasma using PCR-based methods (tested negative).
Commonly misidentified lines (See ICLAC register)	None.

Animals and other organisms

Policy information about studies involving animals; ARRIVE guidelines recommended for reporting animal research

Laboratory animals	Mixed sexes of Asc-/-, Caspase1-/-, IL-1bK133R/K133R, Nlrp3-/-, IL-1b-/- mice on a C57BL/6J background aged between 7-12 weeks were used to generate bone marrow derived cells. For in vivo studies WT controls and IL-1bK133R/K133R were age and sex-matched. These mice were either generated on a C57BL/6J background (Nlrp3-/-) or using strain 129 embryonic stem cells (Caspase-1-/-, Asc-/-) followed by backcrossing onto a C57BL/6J background for at least 10 generations. Il1bK133R/K133R mice harboring a lysine to arginine mutation at amino acid position 133 of IL-1b were generated on a C57BL/6J background.
Wild animals	No wild animals were used.
Field-collected samples	No field collected samples were used.

Ethics oversight	The WEHI Animal Ethics Committee approved all experiments in accordance with the NHMRC Australian code for the care and use of animals for scientific purposes.

Note that full information on the approval of the study protocol must also be provided in the manuscript.

Flow Cytometry

Plots

Confirm that:

- [x] The axis labels state the marker and fluorochrome used (e.g. CD4-FITC).
- [x] The axis scales are clearly visible. Include numbers along axes only for bottom left plot of group (a 'group' is an analysis of identical markers).
- [x] All plots are contour plots with outliers or pseudocolor plots.
- [x] A numerical value for number of cells or percentage (with statistics) is provided.

Methodology

Sample preparation	No flow cytometry plots are provided as data, with the exception of example plots used to describe gating strategy. To evaluate 293T cell viability, cells were harvested and propidium iodide (1-2 µg/ml, PI) uptake measured by flow cytometric analysis on a FACS Calibur instrument and Cell quest software (BD Biosciences). FACs data was analyzed using WEASEL version 2.7 software available at; https://frankbattye.com.au/Weasel/index.html. Viable (PI-) neutrophils (CD11b+ Ly6G+) were sorted from bone marrow using a BD FACSAria III sorter (WEHI).
Instrument	BD FACSCalibur and BD FACSAria III sorter.
Software	Data collected using Cell quest software (BD Biosciences) and analyzed using WEASEL version 2.7 software available at https://frankbattye.com.au/Weasel/index.html, or FlowJo 10.2
Cell population abundance	For in vitro assays – homogeneous cell lines (i.e. 293T) or highly homogeneous bulk bone marrow derived macrophages or neutrophil (isolated) cultures were used in assays, so population abundance is not applicable.
Gating strategy	Provided as Supplemental Figure 6F; Granular leukocyte (P1) single cells (P2) were gated on and viable (propidium iodide negative) neutrophils (Lys6G positive) were sorted (P3) from these.

- [x] Tick this box to confirm that a figure exemplifying the gating strategy is provided in the Supplementary Information.
